# A case report of acute fibrinous and organizing pneumonia

**DOI:** 10.1097/MD.0000000000018140

**Published:** 2019-12-10

**Authors:** Kaige Wang, Xinmiao Du, Qian Wu, Deyun Cheng

**Affiliations:** a Department of Respiratory and Critical Care Medicine; b Department of Pathology, West China Hospital of Sichuan University, Chengdu, Sichuan 610041, China.

**Keywords:** acute fibrinous, corticosteroids, organizing pneumonia, pneumonia

## Abstract

**Rationale:**

Acute fibrinous and organizing pneumonia (AFOP) is a newly evolving rare non-infectious lung pathology, characterized by intra-alveolar fibrin balls on histology. It is usually difficult to be diagnosed and mistaken for other lung diseases.

**Patient concerns:**

In this article, an interesting case about a male patient with a 15-day history of high-grade fever, chills, and no productive cough was presented. He was misdiagnosed as the lung infection early, but exhibited no response to the antibiotic therapy.

**Diagnosis:**

The diagnosis of AFOP was determined by the lung biopsy and pathology.

**Interventions:**

With the diagnosis of AFOP, all antibiotics were discontinued, and 40 mg methylprednisolone daily was given intravenously.

**Outcomes:**

The patient responded well to the treatment with steroids.

**Lessons:**

AFOP is a rare lung disease characterized by bilateral basilar infiltrates and histological findings of organizing pneumonia and intra-alveolar fibrin in the form of “fibrin balls”. Lung biopsy and histopathology were the most important diagnostic methods for the AFOP. Glucocorticoid was an effective drug for the treatment. Subacute patients of AFOP have excellent prognosis with corticosteroids.

## 1 Introduction

Acute fibrinous and organizing pneumonia (AFOP), as a rare disease, is characterized by the deposition of fibrin in alveoli in a patchy pattern, which will result in remarkable severity and mortality. However, the clinical manifestation of AFOP lacks specificity. Two forms of the disease are described: a severe form causing rapid respiratory failure, and a sub-acute form with a better outcome. In most cases, AFOP may be associated with many factors, such as the lung infection, autoimmune diseases, and occupational or chemicals exposure. Idiopathic AFOP is uncommon. In this report, the case of a male patient diagnosed as idiopathic AFOP who was misdiagnosed as community acquired pneumonia (CAP) early, was presented. The patient recovered under the treatment with steroids. Patient has provided informed consent for publication of the case.

## 2 Case report

A 59-year-old man, with no significant medical history, was admitted to our hospital with a 15-day history of high-grade fever, chills, and no productive cough. He reported no weight loss, night sweats, pet contact history, and recent travels. In addition, there was no history of poisoning or exposure to any pets and dusty environmental conditions. But a smoking history of 5 pack years was reported. Before admission to our hospital, the X-ray examination on the chest showed patchy exudation and consolidation. Then he received a 13-day course of the antibiotics therapy (Azithromycin, Cefoperazone and sulbactam, Meropenem). However, he did not recover and was transferred to our hospital for further treatment.

**Figure 1:**
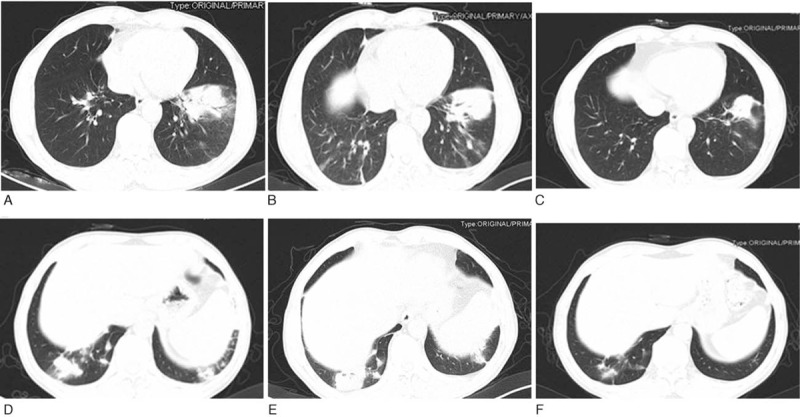
The thoracic CT (A and D) showed bilateral lower lobe consolidation, ground glass opacification and nodules at the time of the administration (day 1). The thoracic CT (B and E) showed no improvement after antibiotic treatment (day 15); left lobe consolidation was unchanged but there were more reticular pattern in the lower lobe of both lungs. The thoracic CT (C and F) showed resorption of the bilateral lower lobe consolidation and significant improvement of opacification 20 days later after the start of glucocorticoid therapy (day 41) compared with the previous scans.

On initial examination, he was febrile (41 °C). The blood pressure, heart rate and respiratory rate were 110/80 millimeters of mercury (mm Hg), 102 beats per minute and 23 breaths per minute, respectively. On physical examination, the lung auscultation revealed the coarse breath sounds with fine crackles in the lower lung zones; while other physical examinations did not exhibit any abnormity. Laboratory testing showed the white cell counts of 13,000 cells per microliter of blood, with neutrophil ratio of 88%. The C reactive protein was elevated to 160 mg/L. Her arterial blood gas analysis demonstrated pH of 7.44, pCO_2_ of 42 mm Hg, and pO_2_ of 76 mm Hg (FiO_2_ = 30%). The thoracic computerized tomography (CT) upon admission showed bilateral lower lobe consolidation, reticulation, and nodules in the left lower lobe (Fig. 1A and D). Blood cultures, sputum cultures, and sputum acid-fast bacilli showed no organisms. Additional tests including mycoplasma chlamydia antibodies, urine legionella antigen, cryptococcal antigen, cytomegalovirus-DNA, Epstein-Barr virus-DNA, procalcitonin, G-test, antinuclear antibodies, extractable nuclear antigens, anti-neutrophil cytoplasmic antibody, glomerular basement membrane antibody, cyclic citrullinated peptide IgG and IgA, creatine phosphokinase, tumor markers (CEA, NSE, CYFRA 211, CA 125, CA153, CA 199), and Human immunodeficiency virus antibodies were all negative. Flexible bronchoscopy followed by bronchoalveolar lavage (BAL) was performed and revealed no endobronchial lesion. BAL specimen did not show any malignant cells. BAL cultures and molecular diagnostic test for tuberculosis were negative; while BAL galactomannan was insignificant. Therefore, the patient was diagnosed as hospital acquired pneumonia initially and started to take 0.5 g imipenem/cilastatin once every 8 hours, and 1 g vancomycin once every 12 hours. On the 6th day of hospitalization, imipenem/cilastatin was switched to moxifloxacin (400 mg daily). On the 11th day of admission, moxifloxacin was exchanged by panipenem/betamipron (0.5 g once every 8 hours).

**Figure 2:**
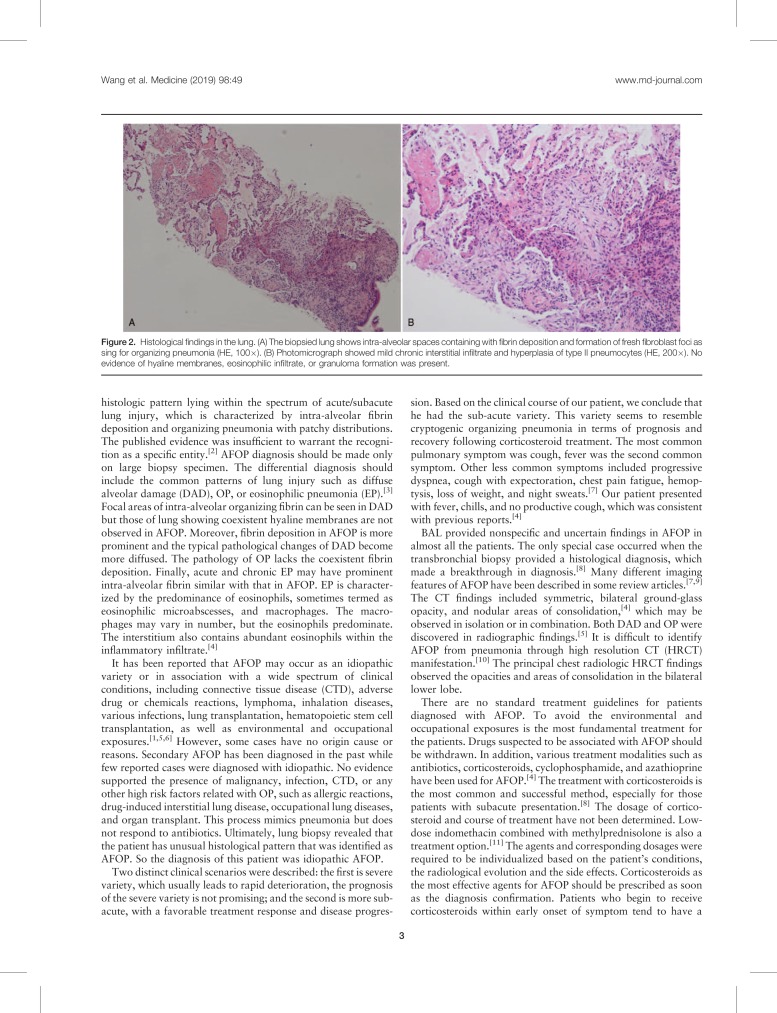
Histological findings in the lung. (A) The biopsied lung shows intra-alveolar spaces containing with fibrin deposition and formation of fresh fibroblast foci as sing for organizing pneumonia (HE, 100×). (B) Photomicrograph showed mild chronic interstitial infiltrate and hyperplasia of type II pneumocytes (HE, 200×). No evidence of hyaline membranes, eosinophilic infiltrate, or granuloma formation was present.

However, his symptoms did not respond to above antibiotics therapy. On the 15th day of hospitalization, pulmonary CT (Fig. 1B and E) scan showed no changes of the lung consolidation, but more reticular patterns in the lower lobe of both lungs. The CT-guided percutaneous needle lung biopsy was performed next. Specimens of the lung biopsy from the left lower lobe were sent for pathology, microbiology, and virology. The pathology (Fig. 2) demonstrated intra-alveolar spaces containing fibrin deposition (so-called fibrin balls) and organizing pneumonia (OP). Mild chronic interstitial infiltrate and hyperplasia of type II pneumocytes were also shown. Neutrophil, eosinophilic infiltration, and hyaline membranes were absent. Immunohistochemistry was listed as follows: IgG4(−), special staining: PAS(−), D-PAS(−), Gomori methenamine silver staining(−), and Perls stain(−). Virology and pathological tissue culture were negative. No granulomas were observed. Those findings were consistent with the symptoms of AFOP.

With the diagnosis of AFOP, all antibiotics were discontinued, and 40 mg methylprednisolone daily was given intravenously. Four days later, fever, chills, and cough all disappeared. The white cell count decreased from 12,000 to 8000 cells per microliter of blood. The patient was discharged home, who had been hospitalized for 21 days with oral prednisone (50 mg daily). Repeated pulmonary CT (Fig. 1C and F) scan performed 20 days later after discharge showed decrease in the appearance of bilateral lower lung consolidation. The patient was symptom-free at follow up after 1 month. He tolerated the steroids well without any obvious adverse reactions. He is currently on tapering prednisolone and on regular follow-up.

## 3 Discussion

AFOP was first reported by Beasley and his colleagues in detail as an unusual type of acute lung injury in 2002. It has been reported in all age groups with average age of 62 years. Males are more commonly affected.^[1]^ In 2013, the updated classification of the idiopathic interstitial pneumonias described AFOP as a rare histologic pattern lying within the spectrum of acute/subacute lung injury, which is characterized by intra-alveolar fibrin deposition and organizing pneumonia with patchy distributions. The published evidence was insufficient to warrant the recognition as a specific entity.^[2]^ AFOP diagnosis should be made only on large biopsy specimen. The differential diagnosis should include the common patterns of lung injury such as diffuse alveolar damage (DAD), OP, or eosinophilic pneumonia (EP).^[3]^ Focal areas of intra-alveolar organizing fibrin can be seen in DAD but those of lung showing coexistent hyaline membranes are not observed in AFOP. Moreover, fibrin deposition in AFOP is more prominent and the typical pathological changes of DAD become more diffused. The pathology of OP lacks the coexistent fibrin deposition. Finally, acute and chronic EP may have prominent intra-alveolar fibrin similar with that in AFOP. EP is characterized by the predominance of eosinophils, sometimes termed as eosinophilic microabscesses, and macrophages. The macrophages may vary in number, but the eosinophils predominate. The interstitium also contains abundant eosinophils within the inflammatory infiltrate.^[4]^

It has been reported that AFOP may occur as an idiopathic variety or in association with a wide spectrum of clinical conditions, including connective tissue disease (CTD), adverse drug or chemicals reactions, lymphoma, inhalation diseases, various infections, lung transplantation, hematopoietic stem cell transplantation, as well as environmental and occupational exposures.^[1,5,6]^ However, some cases have no origin cause or reasons. Secondary AFOP has been diagnosed in the past while few reported cases were diagnosed with idiopathic. No evidence supported the presence of malignancy, infection, CTD, or any other high risk factors related with OP, such as allergic reactions, drug-induced interstitial lung disease, occupational lung diseases, and organ transplant. This process mimics pneumonia but does not respond to antibiotics. Ultimately, lung biopsy revealed that the patient has unusual histological pattern that was identified as AFOP. So the diagnosis of this patient was idiopathic AFOP.

Two distinct clinical scenarios were described: the first is severe variety, which usually leads to rapid deterioration, the prognosis of the severe variety is not promising; and the second is more sub-acute, with a favorable treatment response and disease progression. Based on the clinical course of our patient, we conclude that he had the sub-acute variety. This variety seems to resemble cryptogenic organizing pneumonia in terms of prognosis and recovery following corticosteroid treatment. The most common pulmonary symptom was cough, fever was the second common symptom. Other less common symptoms included progressive dyspnea, cough with expectoration, chest pain fatigue, hemoptysis, loss of weight, and night sweats.^[7]^ Our patient presented with fever, chills, and no productive cough, which was consistent with previous reports.^[4]^

BAL provided nonspecific and uncertain findings in AFOP in almost all the patients. The only special case occurred when the transbronchial biopsy provided a histological diagnosis, which made a breakthrough in diagnosis.^[8]^ Many different imaging features of AFOP have been described in some review articles.^[7,9]^ The CT findings included symmetric, bilateral ground-glass opacity, and nodular areas of consolidation,^[4]^ which may be observed in isolation or in combination. Both DAD and OP were discovered in radiographic findings.^[5]^ It is difficult to identify AFOP from pneumonia through high resolution CT (HRCT) manifestation.^[10]^ The principal chest radiologic HRCT findings observed the opacities and areas of consolidation in the bilateral lower lobe.

There are no standard treatment guidelines for patients diagnosed with AFOP. To avoid the environmental and occupational exposures is the most fundamental treatment for the patients. Drugs suspected to be associated with AFOP should be withdrawn. In addition, various treatment modalities such as antibiotics, corticosteroids, cyclophosphamide, and azathioprine have been used for AFOP.^[4]^ The treatment with corticosteroids is the most common and successful method, especially for those patients with subacute presentation.^[8]^ The dosage of corticosteroid and course of treatment have not been determined. Low-dose indomethacin combined with methylprednisolone is also a treatment option.^[11]^ The agents and corresponding dosages were required to be individualized based on the patient's conditions, the radiological evolution and the side effects. Corticosteroids as the most effective agents for AFOP should be prescribed as soon as the diagnosis confirmation. Patients who begin to receive corticosteroids within early onset of symptom tend to have a better prognosis than those who do not. Our patient presented a clinical manifestation of CAP, but no response to the standard antibiotic therapy. After diagnosed with AFOP, the treatment with 40 mg methylprednisolone daily was given in early stage. There was a dramatic and remarkable clinical response. After 20 days treatment with the oral prednisone, the imaging findings demonstrated the patient recovered obviously.

In conclusion, AFOP is a rare lung disease, characterized by bilateral basilar infiltrates and histological findings of OP and intra-alveolar fibrin in the form of “fibrin balls.” AFOP should be considered different from the CAP, because of the nonresponse to standard antibiotic therapy. Lung biopsy and histopathology, which were the most important diagnostic methods, should be performed. The treatment with glucocorticoids is an effective drug method. Subacute patients of AFOP exhibited excellent prognosis by the treatment with corticosteroids.

## Acknowledgment

We thank the patient and his family for their kind cooperation.

## Author contributions

**Conceptualization:** Kaige Wang.

**Data curation:** Kaige Wang, Xinmiao Du and Qian Wu.

**Formal analysis:** Deyun Cheng.

**Funding acquisition:** Deyun Cheng.

**Investigation:** Kaige Wang and Xinmiao Du.

**Methodology:** Deyun Cheng.

**Project administration:** Kaige Wang and Deyun Cheng.

**Resources:** Qian Wu.

**Writing – original draft:** Kaige Wang.

**Writing – review & editing:** Xinmiao Du and Deyun Cheng.
